# Predictive value of PET metabolic parameters for occult lymph node metastases in PET/CT defined node-negative patients with advanced epithelial ovarian cancer

**DOI:** 10.1038/s41598-023-36640-0

**Published:** 2023-06-09

**Authors:** Bing Xue, Xihai Wang

**Affiliations:** 1 Department of Nuclear Medicine, The Fourth Affiliated Hospital of China Medical University, Shenyang, 110032 China; 2 Department of Radiology, Shengjing Hospital of China Medical University, Shenyang, 110004 China

**Keywords:** Positron-emission tomography, Gynaecological cancer

## Abstract

Accurate lymph node metastasis (LNM) prediction is crucial for patients with advanced epithelial ovarian cancer (AEOC) since it guides the decisions about lymphadenectomy. Previous studies have shown that occult lymph node metastasis (OLNM) is common in AEOC. The objective of our study is to quantitatively assess the probability of occult lymph node metastasis defined by ^18^F-Fluorodeoxyglucose PET/CT in AEOC and explore relationship between OLNM and PET metabolic parameters. The patients with pathologically confirmed AEOC who underwent PET/CT for preoperative staging at our institute were reviewed. Univariate and multivariate analysis were performed to evaluate the predictive value of PET/CT-related metabolic parameters for OLNM. The result of our study showed metastatic TLG index had a better diagnostic performance than other PET/CT-related metabolic parameters. Two variables were independently and significantly associated with OLNM in multivariate analysis: metastatic TLG index and primary tumor location. The logistic model combining metastatic TLG index, primary tumor location, and CA125 might be a promising tool to effectively predict the individualized possibility of OLNM for AEOC patients.

## Introduction

Ovarian carcinoma is one of the deadliest gynecologic cancers because the majority of patients are diagnosed with advanced-stage (stage III and IV) according to the International Federation of Gynecology and Obstetrics (FIGO) staging classification^1^. Epithelial ovarian cancer (EOC) is the most crucial histological subtype, accounting for approximately 70% of ovarian cancers^2,3^. Lymph node (LN) status has an important impact on the FIGO stage of EOC^4,5^. Although debulking surgery is the standard treatment of AEOC, pelvic and para-aortic lymphadenectomy is not necessary for all patients^6^. Studies have shown no benefit in overall survival or progression-free survival between cytoreductive surgery with and without pelvic and para-aortic lymphadenectomy, suggesting that lymphadenectomy should be omitted for those patients without LNM^7,8^. Identification of metastatic LN in patients with AEOC before debulking surgery is important as it guides decisions about lymphadenectomy.

The frequency of LNM observed in EOC is up to 75% of patients with stage III–IV and 25% of patients with stage I–II^8,9^. LNM has been evaluated using several non-invasive modalities of preoperative imaging, such as computed tomography (CT), magnetic resonance imaging (MRI), and 2-[^18^F] fluoro-2-deoxy-D-glucose (^18^F-FDG) PET/CT^10–13^. Clinical OLNM is defined as no suspicion of lymph node involvement on CT or PET images^14^. In prior studies, OLNM was mainly defined according to CT modality. However, PET/CT, which can provide the functional and anatomical information of malignancy, is considered the most accurate LNM staging modality^15–17^. Although preoperative PET/CT showed higher diagnostic accuracy than CT in detecting pelvic and para-aortic LNM in AEOC, the sensitivity of PET/CT was unsatisfactory, ranging from 26.7 to 87.6% ^18–20^. Low diagnostic sensitivity of PET/CT indicated clinical OLNM was common for AEOC, which accounted for approximately 20–80% of patients^10,16,18^.

Therefore, the challenge is to distinguish the patients with OLNM who should have a lymphadenectomy from the patients with “no suspicious lymph nodes” who should not have lymphadenectomy. Previous studies have demonstrated that certain PET/CT parameters, such as standardized uptake value (SUV), metabolic tumor volume (MTV), and total lesion glycolysis (TLG), were significant prognostic factors for AEOC^21–25^. The SUV is a ratio of radioactivity concentration in tissue at a point in time divided by the injected dose of radioactivity per kilogram of the patient’s weight, which is a simple way of determining activity in PET imaging. MTV refers to the metabolically active volume of the tumor segmented using FDG PET, and has been shown to be useful in predicting patient outcome and in assessing treatment response. However, there was no study to evaluate the correlation between PET/CT-related parameters and LNM, especially OLNM. Previous studies have demonstrated that hypermetabolic LN on PET/CT, initial CA125 ≥ 500, and initial peritoneal cancer index (PCI) ≥ 10 were independently and significantly associated with pelvic and/or para-aortic LNM^26,27^. Recently, many researchers have focused on radiomics features of PET/CT or contrast-enhanced CT in predicting LNM in AEOC, among which LNM assessment has been proven to have a certain significant improvement^28,29^. However, the manually time-consuming delineation of region of interesting (ROI) and complex models limit its clinical application. The objective of our study is to evaluate the relationship between PET/CT-related parameters and OLNM and construct a simple model to predict OLNM.

## Materials and methods

### Patients

The study was conducted in accordance with the Declaration of Helsinki (as revised in 2013). This retrospective study was approved by the medical ethics committee of Shengjing Hospital of China Medical University (2020PS374K). Informed consent was not required by medical ethics committee of Shengjing Hospital of China Medical University due to the retrospective nature of the study.

From January 2013 to December 2022, a total of 777 consecutive patients with pathologically confirmed EOC who underwent ^18^F-FDG PET/CT for preoperative staging at our institute were reviewed. Inclusion criteria were as follows: (1) received debulking surgery with pelvic and para-aortic lymphadenectomy; (2) postoperative pathological examination confirmed stage III or IV EOC with definite LN status; (3) PET/CT scan was performed within one month before surgery. The exclusion criteria included the following: (1) received any neoadjuvant chemotherapy (NAC) before surgery; (2) poor image quality or image data loss; (3) negative uptake of the primary or metastatic lesions in PET/CT images (SUVmax < 2.5).

The PET/CT images of patients who met the inclusion criteria were evaluated by two radiologists (with more than 5 years of experience in abdominal imaging of PET/CT images) to confirm presence of positive LN on PET/CT images. Further analysis was performed for the patients with no positive LN on PET/CT, and those patients were categorized into two groups based on postoperative pathological findings: those with LNM (OLNM) and those without any LN involvement (NLNM). The patients with positive LN on PET/CT images were not further analyzed.

**Figure 1:**
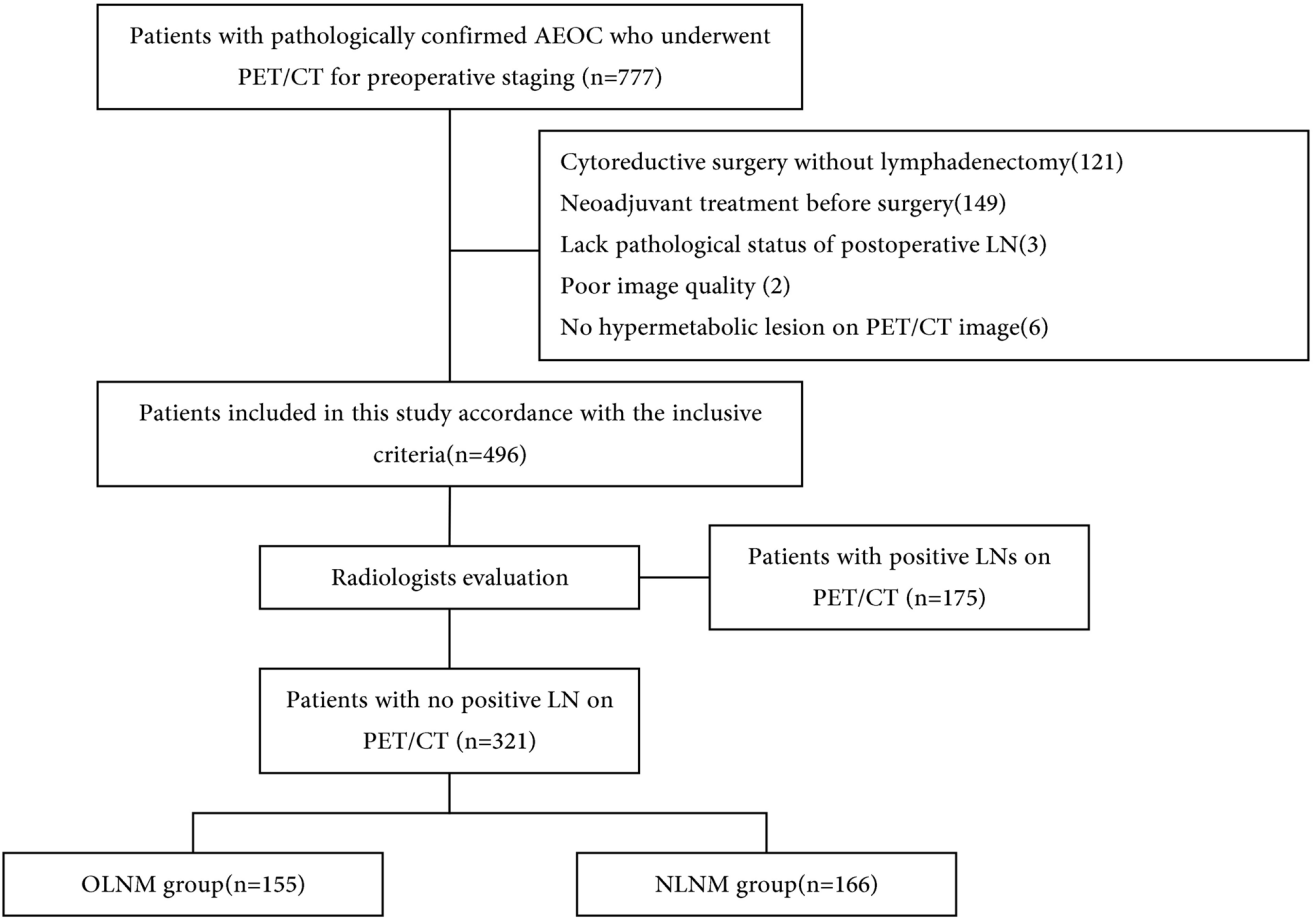
Flow chart of patient selection.

The flowchart of patient selection is shown in Fig. 1.

Clinical data such as age, menopause status, CA125 and so on were collected from the hospital information system of our hospital. The ascites and location of primary tumor were evaluated by previous radiologists on PET/CT scans. The ascitic volume was divided into two categories: small (< 1500 ml) and large amount of ascitic (≥ 1500 ml), according to ascetic volume assessed on CT component of PET/CT^30^. The classification of ascites volume is based on our clinical experience and previous studies^31^. FDG avid in one side or two side tubo-ovarian was defined as unilateral or bilateral involvement.

### LN status assessment

**Figure 2:**
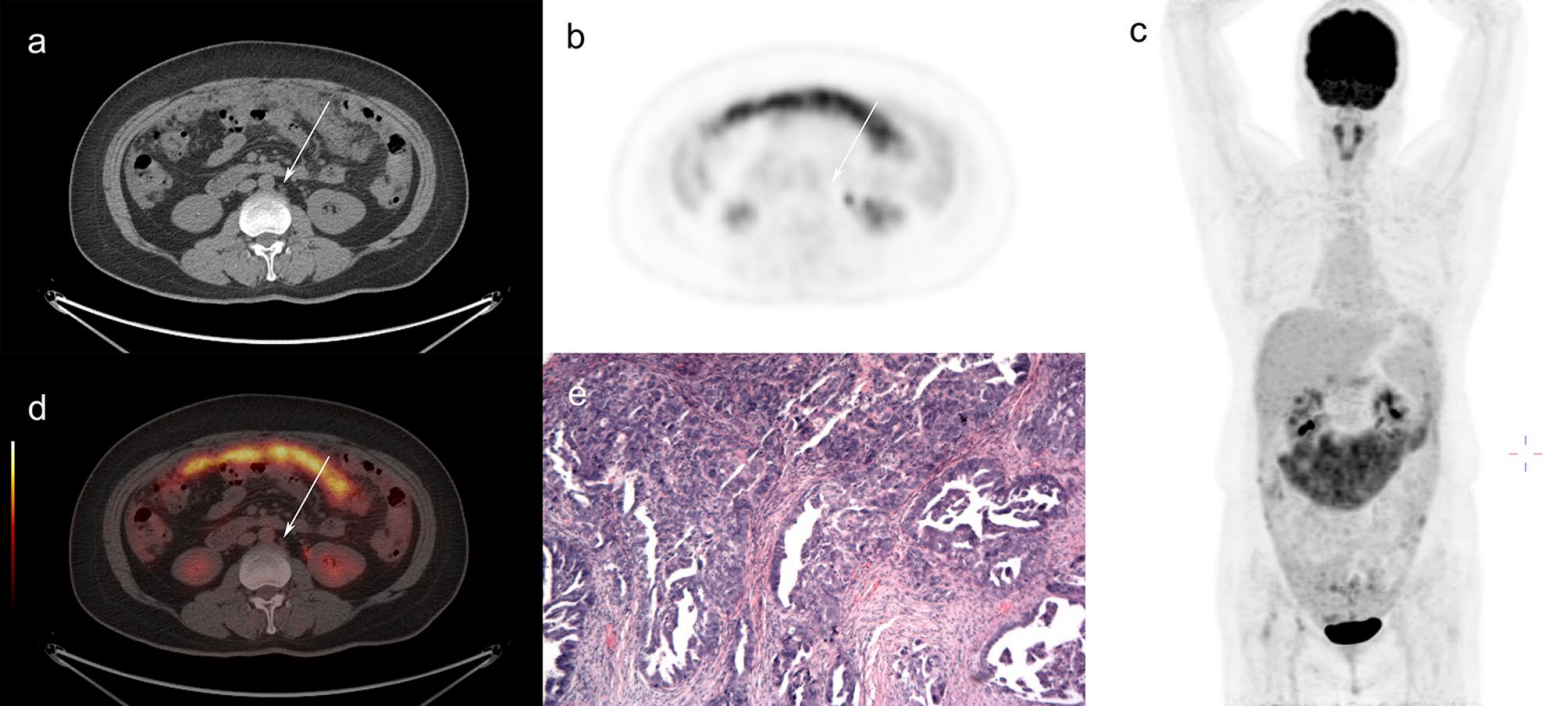
A 42-year-old female with pathologically diagnosed high-grade serous carcinoma. Pelvic and para-aortic lymph node metastasis were confirmed by postoperative pathological results. There were small para-aortic lymph nodes presented in the lymph draining area on CT scan (**a**). However, no FDG avid in those lymph nodes on maximum intensity projection (**b**, **c**) and fused PET/CT (**d**) images. The postoperative pathological results confirmed lymph node metastasis of high-grade serous carcinoma, HE staining, magnification 100× (**e**).The metastatic MTV and TLG index of this patient was 96.2% and 97.5%, respectively.

In our study, the procedures for LN dissection included pelvic lymphadenectomy and para-aortic lymphadenectomy. The extent of LN dissection included seven subregions: para-aortic, common iliac, lateral sacral, external iliac, internal iliac (hypogastric), obturator, and inguinal. The LN dissection and postoperative pathological results were classified by subregion. Our study defined LNM as confirmed involvement of one or more of the above subregions based on postoperative pathological results. The preoperative LN status on PET/CT images of enrolled patients was evaluated by two radiologists according to the seven subregions (Fig. 2) . Inconsistent results were resolved through consultation by the two radiologists. OLNM was defined as postoperative pathological confirmation of LNM with no suspicion of LN involvement on either CT (short-axis diameter < 1 cm) or PET (SUVmax < 2.5).

### ^18^F-FDG PET/CT image acquisition

Whole-body ^18^F-FDG PET/CT scans were performed using a dedicated PET/CT scanner (Discovery 690, GE Healthcare, Milwaukee, WI) within one month before any treatment. Patients fasted for at least 6 h and were injected with 3.7 MBq/kg ± 10% of body weight ^18^F-FDG. The fasting serum glucose and insulin injection were strictly controlled. Patients with uncontrolled diabetes or hyperglycemia may require rescheduling of the scan to avoid inaccurate results. Then the acquisition was performed after approximately 60 min. The 3D ordered subset expectation maximization (OSEM) algorithm with attenuation correction was used for the reconstruction of the PET image. The voxel sizes of the reconstructed PET image were 3.65 × 3.65 × 3.27 mm^3^, and the matrix size was 192 × 192. CT scans (109 mA, 140 kV) were performed before the PET scan with a matrix size of 512 × 512 and a voxel size of 0.98 × 0.98 × 3.75 mm^3^.

### Measurement of PET metabolic parameters

Finally, PET/CT images were imported into 3D Slicer software (version 4.8.1, https://www.slicer.org/) to extract PET metabolic parameters. The PETTumorSegmentation extension within 3D Slicer was used to delineate primary and metastatic lesions, providing an editor-effect for semi-automated segmentation of tumors and hot lymph nodes in PET scans ^32^. Multiple areas were selected as ROI. To avoid including physiologic uptake in the volume of interest, a combined CT and PET scan reading was performed. PET-IndiC, another extension of 3D Slicer, was used to calculate PET-related metabolic parameters. The following parameters were measured in primary and all lesions: SUVmean, SUVmax, MTV, TLG, and SUVpeak. The SUVmax was determined as the maximum value in multiple ROIs. MTV or TLG was calculated as the sum of each ROI, while SUVmean and SUVpeak were determined as the mean of each ROI. The MTV and TLG of metastatic lesions were calculated by subtracting the values of primary lesions from those of all lesions. The proportion of MTV and TLG of metastatic lesions in relation to all lesions were defined as the metastatic MTV index and metastatic TLG index, respectively.

$${\text{Metastatic}}\;{\text{MTV}}\;{\text{index}} = \frac{{{\text{MTV}}\;{\text{metastases}}}}{{{\text{MTV}}\;{\text{all}}\;{\text{lesions}}}} \times 100\%$$

$${\text{Metastatic}}\;{\text{TLG}}\;{\text{index}} = \frac{{{\text{TLG}}\;{\text{metastases}}}}{{{\text{TLG}}\;{\text{all}}\;{\text{lesions}}}} \times 100\%$$

### Statistical analysis

Student t-tests or Mann–Whitney U tests were used for continuous variables, and Chi-squared tests and exact Fisher tests were applied for categorical variables between two groups. The association between variables and OLNM was evaluated by univariate logistic regression analysis. The features with a *p* value < 0.05 in the univariate logistic regression analysis were selected for multivariate logistic regression analysis. The multivariate logistic regression analysis with the optimal subset method was used to assess the best combination of variables that were independently associated with OLNM. The ROC curve was used to assess the performance of the final logistic model. All analysis and calculations were done with R (version 4.0.5, www.Rproject.org). A *p* value < 0.05 was considered statistically significant.

### Ethics approval and content to participate

Ethics approval was granted by the ethics committee of the Shengjing Hospital of China Medical University.

## Result

### Patient selection and clinical characteristics

Our study included 496 patients with primary AEOC who met all inclusion criteria. Among them, 175 patients showing positive LNs on PET/CT images were excluded. Finally, 321 patients who were evaluated by radiologists and showed no positive LN on PET/CT images were divided into OLNM group (n = 155) and the NLNM group (n = 166). OLNM accounted for 51.3% of all LNM and 48.3% of negative LN on PET/CT scans. The characteristics of the patients for further analysis are shown in Table 1. In the OLNM group, 48 patients were associated with both pelvic and para-aortic LNM, 86 patients only had pelvic LNM, and 21 patients only had para-aortic LNM. There was no significant difference in the number of LNs resected in cytoreductive surgery, 20.434 ± 10.543 in the NLNM group and 21.987 ± 12.210 in the OLNM group. The age, initial HE4, initial 724, and menopausal state showed no statistically significant between two groups.

**Table 1:** Patients characteristics.

Characteristics	NLNM group n = 166	OLNM group n = 155	*P* value
Age	55.825 ± 9.660	54.245 ± 9.271	0.136
*Ascites*			0.005
Small	111 (66.867%)	79 (50.968%)	
Large	55 (33.133%)	76 (49.032%)	
HE4	524.233 ± 405.548	618.708 ± 409.960	0.039
CA199	67.658 ± 168.730	33.129 ± 81.675	0.019
CA724	43.803 ± 72.517	63.302 ± 91.523	0.036
CA125	1067.805 ± 1264.763	1461.316 ± 1479.211	0.011
*Pathological type*			0.069
HGSOC	136 (81.928%)	139 (89.677%)	
Others	30 (18.072%)	16 (10.323%)	
*Menopausal state*			0.453
No	41 (24.699%)	45 (29.032%)	
Yes	125 (75.301%)	110 (70.968%)	
Resected LN numbers	20.434 ± 10.543	21.987 ± 12.210	0.223
Resected LN stations	0.000 ± 0.000	4.000 ± 4.218	< 0.001
*Location*			0.001
Unilateral	51 (30.723%)	22 (14.194%)	
Bilateral	115 (69.277%)	133 (85.806%)	
Primary lesions SUVmean	6.409 ± 2.603	6.394 ± 4.145	0.969
Primary lesions SUVmax	15.786 ± 7.022	15.267 ± 13.879	0.676
Primary lesions MTV	174.514 ± 163.401	128.518 ± 130.195	0.005
Primary lesions TLG	1196.512 ± 1326.612	821.808 ± 871.071	0.003
Primary lesions SUVpeak	12.936 ± 6.067	11.947 ± 8.602	0.238
All lesions SUVmean	6.193 ± 2.497	5.814 ± 2.079	0.14
All lesions SUVmax	17.497 ± 11.131	17.226 ± 17.489	0.87
ALL lesion MTV	311.310 ± 255.478	385.207 ± 292.735	0.016
ALL lesion TLG	2016.447 ± 2019.289	2347.499 ± 2181.457	0.159
ALL lesion SUVpeak	13.824 ± 6.860	19.437 ± 54.441	0.204
Metastatic lesions MTV	136.792 ± 232.498	251.595 ± 264.120	< 0.001
Metastatic MTV index	0.355 ± 0.351	0.577 ± 0.341	< 0.001
Metastatic lesions TLG	819.936 ± 1512.754	1478.419 ± 1774.855	< 0.001
Metastatic TLG index	0.334 ± 0.351	0.559 ± 0.343	< 0.001

### Predictive performance of PET metabolic parameters

**Figure 3:**
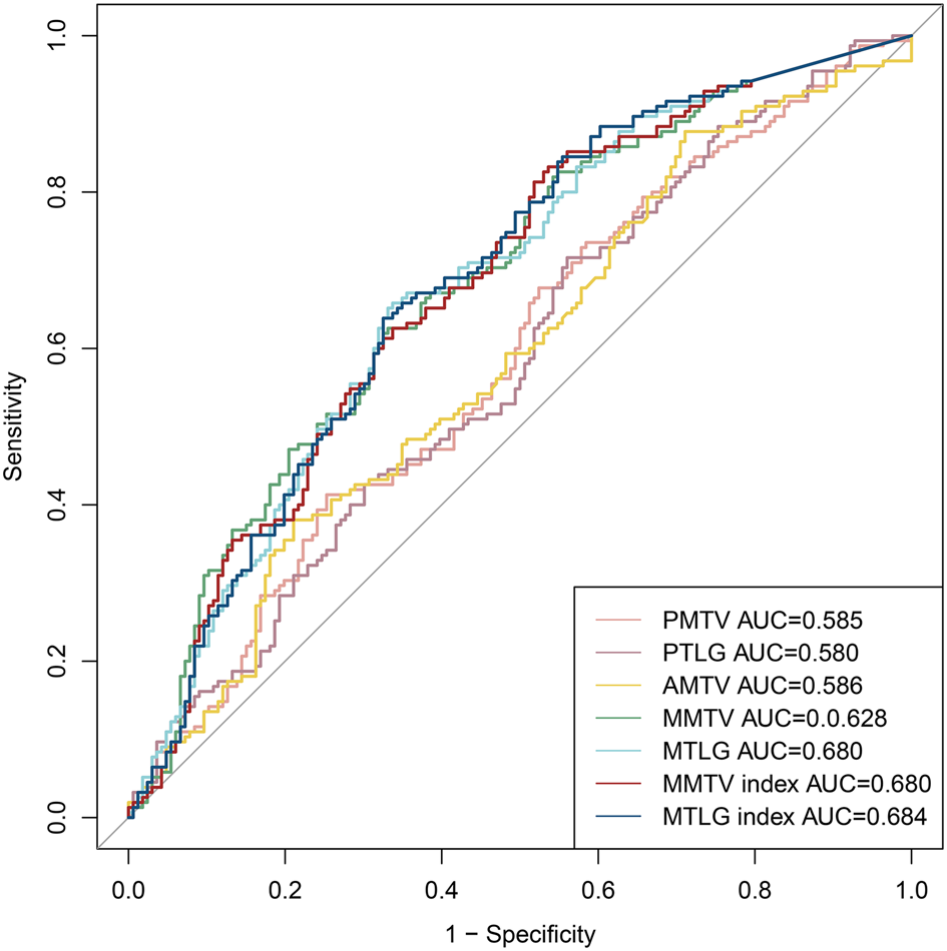
ROC curves of PET-related metabolic parameters for OLNM.

The results of univariate logistic regression analysis of PET-related metabolic parameters showed MTV of primary lesions, TLG of primary lesions, MTV of all lesions, MTV of metastatic lesions, metastatic MTV index, and metastatic TLG index were significantly associated with OLNM. In ROC curve analysis, the AUC of metastatic TLG index (0.684, 95% CI 0.645–0.760) was larger than other PET-related metastatic parameters (Fig. 3). We found severe collinearity between PET-related metabolic parameters with *p* < 0.05 in univariate analysis. The correlation between those parameters was assessed by Pearson correlation analysis (Table 2).

**Table 2:** Pearson correlation of the PET/CT parameters with *p* < 0.05 in univariate analysis.

Variables	Primary lesions MTV	Primary lesions TLG	ALL lesion MTV	Metastatic lesions MTV	Metastatic MTV index	Metastatic lesions TLG	Metastatic TLG index
Primary lesions MTV	1.000	0.849	0.352	− 0.217	− 0.560	− 0.163	− 0.556
Primary lesions TLG	0.849	1.000	0.345	− 0.142	− 0.444	− 0.038	− 0.457
ALL lesion MTV	0.352	0.345	1.000	0.824	0.343	0.802	0.348
Metastatic lesions MTV	− 0.217	− 0.142	0.824	1.000	0.692	0.941	0.695
Metastatic MTV index	− 0.560	− 0.444	0.343	0.692	1.000	0.615	0.990
Metastatic lesions TLG	− 0.163	− 0.038	0.802	0.941	0.615	1.000	0.623
Metastatic TLG index	− 0.556	− 0.457	0.348	0.695	0.990	0.623	1.000

### Risk factors and prediction model

**Figure 4:**
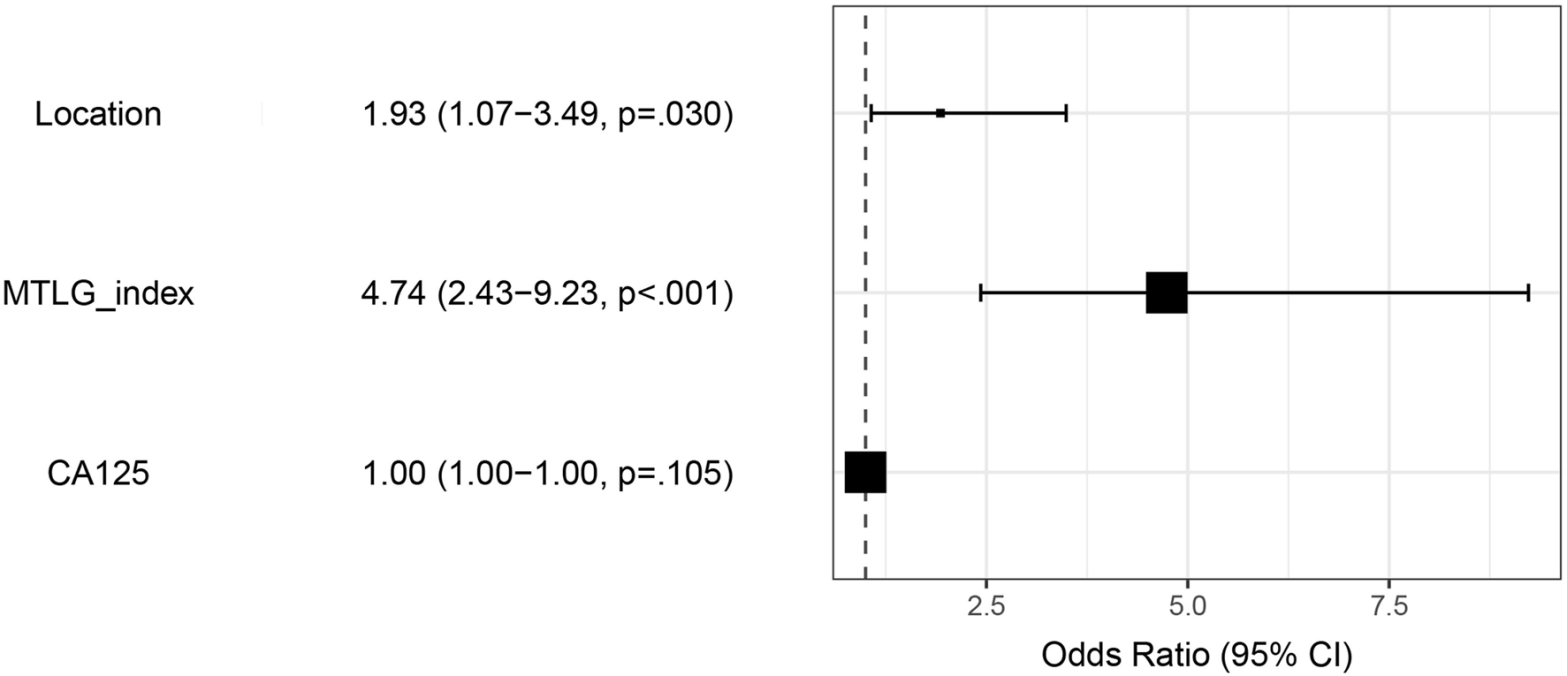
The display of multivariate logistic regression using forest plot.

**Figure 5:**
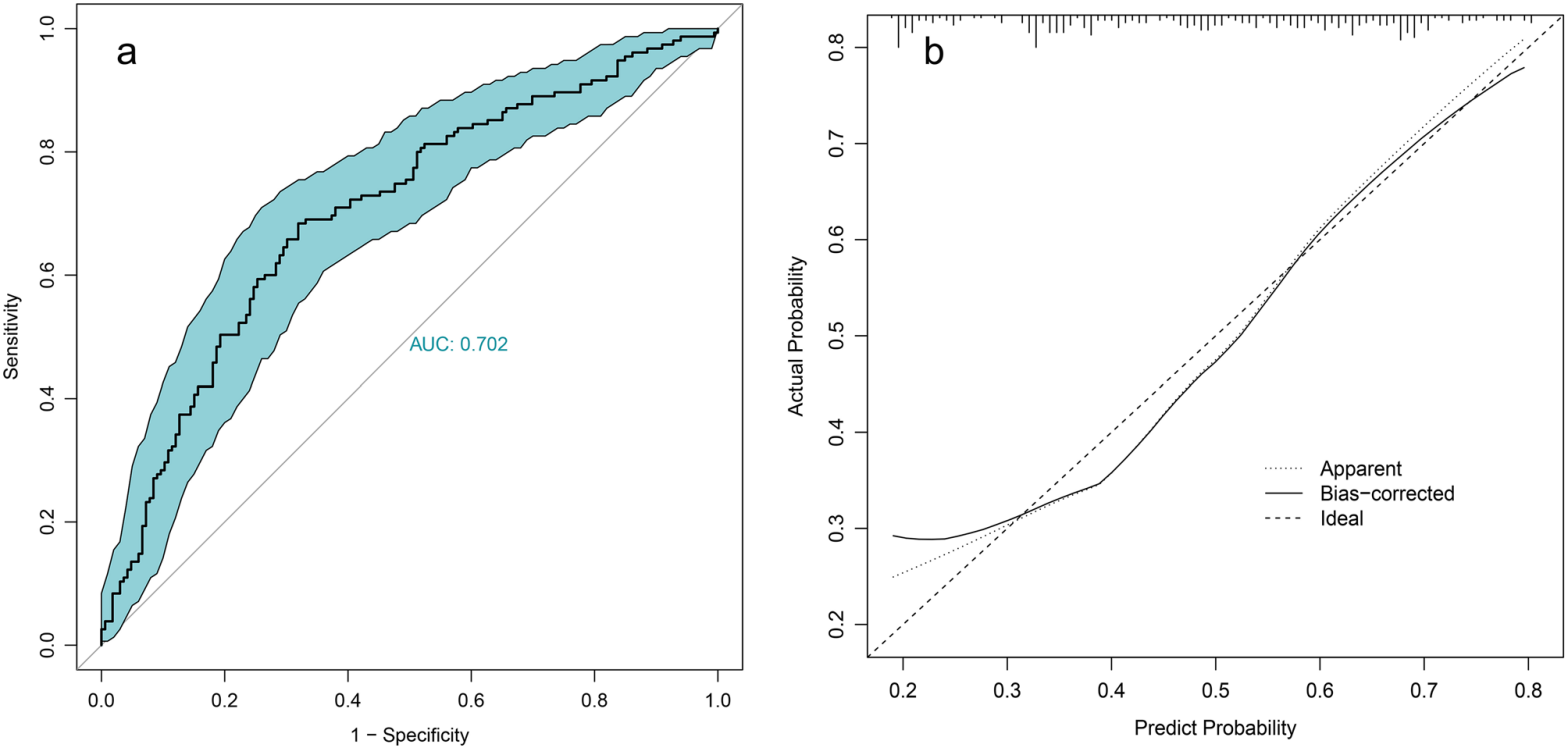
Internal calibration of the final logistic regression model. ROC curve of the logistic regression model (**a**) with bootstrap 1000 times, Calibration curve with the Hosmer–Lemeshow test (**b**).

Among all clinical characteristics, the volume of ascites, CA125, and location of the primary tumor were significantly correlated with a high risk of OLNM (*p* < 0.05). To eliminate the correlation and multiple linearities between the variables, particularly PET-related metabolic parameters, multivariate logistic regression with an optimum subset method was utilized to simplify the financial indexes. The findings from the univariate and multivariate logistic analysis are presented in Table 3. The metastatic TLG index (OR = 4.740, 95% CI 2.453–9.320) and location of primary tumors (OR = 1.930, 95% CI 1.074–3.531) were significantly associated with OLNM (*p* < 0.05) in multivariate logistic analysis. The results are displayed using forest plots (Fig. 4). The ROC-AUC of the final logistic model after bootstrap replicates 1000 times was 0.702 (95% CI 0.645–0.759) (Fig. 5a). The predicted and observed probabilities of OLNM is shown in Fig. 5b and do not differ significantly (*p* = 0.06).

**Table 3:** Univariate and multivariate logistic analysis for OLNM.

Varibles	Univariate analysis		Multivariate analysis	
	OR (95% CI)	*P*	OR (95% CI)	*P*
Age	0.982(0.959,1.005)	0.137		
Ascites (Small/Large)	1.941(1.239,3.059)	0.004		
CA199	0.997(0.995, 0.999)	0.036		
HE4	1.000(0.999,1.000)	0.314		
CA724	1.002(1.000,1.005)	0.053		
CA125	1.000(1.000,1.000)	0.012	1.000(1.000–1.000)	0.104
Menopausal state (Yes/No)	0.802(0.488,1.315)	0.381		
Pathological (HGSOC/Others)	0.521(0.266,0.988)	0.05		
Location (Unilateral/Bilateral)	2.681(1.550,4.761)	0.001	1.930(1.074–3.531)	0.03
Primary lesions SUVmean	0.998(0.931,1.068)	0.968		
Primary lesions SUVmax	0.995(0.970,1.016)	0.673		
Primary lesions MTV	0.997(0.996,0.999)	0.007		
Primary lesions TLG	0.999(0.999,0.999)	0.005		
Primary lesions SUVpeak	0.980(0.942,1.011)	0.264		
All lesions SUVmean	0.928(0.834,1.023)	0.148		
All lesions SUVmax	0.998(0.981,1.014)	0.867		
ALL lesions MTV	1.001(1.000,1.001)	0.019		
ALL lesions TLG	1.000(0.999,1.000)	0.163		
ALL lesions SUVpeak	1.004(0.996,1.025)	0.451		
Metastatic lesions MTV	1.004(1.002,1.007)	< 0.001		
Metastatic MTV index	5.801(3.069,11.19)	< 0.001		
Metastatic lesions TLG	1.001(0.999,1.004)	0.156		
Metastatic TLG index	5.908(3.129,11.39)	< 0.001	4.740(2.453–9.320)	< 0.001

## Discussion

The state of LN in patients with AEOC affects the choice of lymphadenectomy and prognosis^33^. This was the first clinical study to assess occult pelvic and para-aortic LNM in patients with AEOC after cytoreductive surgery. In our study, the proportion of clinical OLNM was high, accounting for 51.3% of all LNM and 48.3% of negative LN on PET/CT scans. The primary tumor location and metastatic TLG index extracted from TLG of metastatic and all lesions were significantly associated with OLNM. The final logistic model could predict OLNM in AEOC before surgery.

The use of systematic lymphadenectomy is controversial for patients with EOC. Although the positive rate of lymph nodes in patients with presumed early-stage EOC was low (appropriately 3–14% detected by systematic lymphadenectomy), LNM in AEOC was high^4,5,34^. LNM was found in 60.9% of all patients in our study, similar to the previous studies^9^. Occult nodal involvements were frequently found in AEOC. In our study, only 48.75% of the cases with LNM had positive lymph nodes on PET/CT scans. The other 51.25% of cases were negative on PET/CT scans. ^18^F-FDG PET/CT is the most accurate modality for preoperative tumor staging and detection of metastatic lymph nodes. A high proportion of LNM is negative in PET/CT scans due to the low spatial resolution of PET-CT images and the low tissue uptake of the ^18^F-FDG. Most of the metastatic lesions, including LNM, tents to uptake less FDG than primary lesions. Furthermore, tumor FDG binding depends on histological type and has a low affinity in mucus and clear cell adenocarcinoma^35^. In addition, the micro-metastases will not be detected due to the low reconstructed spatial resolution of PET. A previous study demonstrated sensitivity for detecting metastatic lesions 4 mm or less in short-axis diameter was 12.5%, and for between 5 and 9 mm was 66.7%^36^. Finally, massive ascites and extensive peritoneal metastasis also limit the detection of LNM on PET/CT scans. In summary, OLNM is frequently observed in AEOC, which leads to the low sensitivity of PET/CT for LNM evaluation.

Many previous studies demonstrated that it was feasible to improve the diagnostic efficiency in LNM by clinical factors, radiological findings, and radiomics features extracted from PET or CT images. A new diagnostic tool based on multivariate analysis, including three variables—pelvic and/or para-aortic LNM on CT PET/CT, initial PCI ≥ 10, and initial CA125 ≥ 500—was proposed^27^. Another study used preoperative radiological scores to predict pelvic and/or para-aortic LNM^28^. Although the ROC-AUC of those prediction models were moderate, the preoperative assessment of PCI/or colon involvement was difficult and ambiguous. Many studies have demonstrated the value of radiomics features in predicting LNM of EOC^28,37^. The radiomics signatures extracted from CT images incorporating LN reports by radiologists could improve the diagnostic efficiency in LNM^37^. However, the calculation of the radiomics features is very complicated and time-consuming. We also found that the evaluation of LNM mainly relied on the subjective assessment of LNM by radiologists in previous studies. It is easy to identify positive LN on PET/CT scans because of hypermetabolism of metastatic LN. Identification of OLNM is critical for improving sensitivity in detecting LNM.

Previous studies have investigated the predictive value of PET metabolic parameters in AEOC^25,27,31^. MTV and TLG were found to be independent prognostic factors for disease progression and overall survival after cytoreductive surgery in patients with EOC^23,25^. In our study, MTV, TLG, metastatic TLG index, and metastatic MTV index were highly associated with OLNM. The metastatic TLG index was the only independent predictive factor in both univariate and multivariate analysis and had a higher predictive value than other PET-related parameters. The metastatic TLG and MTV indexes can quantify EOC's metastatic capacity, which positively correlate with metastatic tumor burden. OLNM, as part of metastatic lesions, was closely associated with soakage and metastasis of malignant ovarian tumors.

The metastatic TLG index had a better AUC (0.684) compared to other PET metabolic parameters. However, combining clinical features can significantly improve the predictive ability of OLNM. In this study, the final multivariate model incorporated CA125, bilateral ovarian involvement, and metastatic TLG index. Bilateral ovarian cancers were found to be associated with a higher risk of OLNM due to increased tumor heterogeneity and invasiveness. Although CA125 was not an independent predictive factor in the multivariate analysis, it plays an important role in ovarian cancer diagnosis and follow-up.

There are several limitations to the present study. Firstly, this study is a retrospective analysis conducted at a single center, which may potentially suffer from inherent selection bias and limit the generalizability of the data. All patients underwent PET/CT scans using the same devices at our hospitals. However, the resolution of the images, administration of a carbohydrate-free diet before scanning, and time for uptake were inevitably different, which may have resulted in heterogeneous results. Further multicenter studies are necessary to confirm the efficiency of the results of this study. Secondly, our study only used PET/CT to predict OLNM. Although PET/CT is the most accurate imaging modality for detecting LNM, a comparison of PET/CT with contrast-enhanced CT or MRI for OLNM will be necessary in further studies. Thirdly, our study focused on patients with negative LN on PET/CT scans. Only the patients with negative reports in PET/CT scans were included in our study. A comparison between OLNM and pathologically confirmed no LNM, regardless of positive LN on PET/CT, will be needed in further studies.

## Conclusions

In conclusion, our study demonstrated that OLNM defined by PET/CT was frequently observed in patients with AEOC. The metastatic TLG index extracted from metastatic and all hot lesions was found to be a significant predictor of OLNM. The logistic model, which combines the metastatic TLG index, primary tumor location, and CA125 is a promising tool for improving the diagnostic accuracy of OLNM. Hence, this simple method may provide valuable information for triaging patients according to simple lymphadenectomy decision rules.

## Data Availability

The datasets used and/or analyzed during the current study are available from the corresponding author on reasonable request.
